# L-Canavanine potentiates the cytotoxicity of doxorubicin and cisplatin in arginine deprived human cancer cells

**DOI:** 10.7717/peerj.1542

**Published:** 2016-01-07

**Authors:** Agustina DR Nurcahyanti, Michael Wink

**Affiliations:** Institute of Pharmacy and Molecular Biotechnology, Heidelberg University, Heidelberg, Germany

**Keywords:** L-canavanine, L-arginine, Doxorubicin, Cisplatin, Argininosuccinate synthase (ASS), Synergy

## Abstract

The non-protein amino acid L-canavanine (L-CAV), an antimetabolite of L-arginine (L-ARG), can alter the 3D conformation of proteins when incorporated into a protein instead of L-ARG. L-CAV inhibits the proliferation of some tumour cells. The deprivation of L-ARG in the culture medium enhances the response of cells to L-CAV. This study aimed to investigate the interaction of L-CAV in combination with the chemotherapeutic drugs, doxorubicin (DOX) or cisplatin (CIS), in cancer cells, especially in the absence of L-ARG. A combination method based on the median-effect principle and mass-action law was used. The following cancer cells were employed: HeLa and Caco-2 cells, overexpressing argininosuccinate synthase (ASS), pancreatic cells (MIA PaCa-2 and BxPC-3) and hepatocellular carcinoma cells (Hep G2 and SK-HEP-1), with down-regulated ASS. When constant and non-constant ratios of L-CAV were combined with DOX and CIS, a synergistic potentiation of cytotoxicity was recorded. Cells expressing high levels of ASS were more sensitive to the treatment as compared to the cells with reduced ASS levels. Overall, this study may provide a new approach to targeting some cancer cells with L-CAV in combination with DNA-targeting drugs such as DOX and CIS, especially those cells which overexpress ASS, such as human cervical and colorectal carcinoma cells.

## Introduction

Twenty-two protein amino acids (including selenocysteine and pyrrolysine) are common to all organisms. However, more than 700 amino acids exist in nature, especially in plants, which are structural analogues of protein amino acids and termed non-protein amino acids (NPAA). Some of them, such as those from the legume genus *Lathyrus*, are toxic and cause neurological disorders in both man and animals (Bell, 2003; Wink & Wyk, 2008). Many NPAAs act as antimetabolites when integrated into proteins, because some aminoacyl tRNA synthetases do not discriminate between a protein amino acid and its corresponding NPAA. When incorporated into a protein, very often such a protein will assume a different 3D structure than the original protein and therefore loses its bioactivity. This property provides plants, which produce NPAAs, with a defence mechanism against herbivores, microbes, and viruses, and competing plants (Bell, 2003). Some NPAAs, such as L-canavanine, L-canaline, β-aminopropionitrile (BAPN), azaserine, and mimosine are able to inhibit the growth of some cancer cell lines (Bondareva et al., 2009; Chung et al., 2012; Jang et al., 2002; Lyons, Sant & Christopherson, 1990; Rosenkranz & Wink, 2007; Rosenthal, 1997).

L-canavanine (L-CAV), first isolated in 1929 from *Canavalia ensiformis* (Fabaceae), is a structural homologue of L-arginine (L-ARG). When L-CAV is incorporated into a protein instead of L-ARG during ribosomal protein biosynthesis, the protein structure and function are altered, often leading to an inhibition of cell growth and causing cellular death (Bence, Adams & Crooks, 2003). Some studies have revealed that L-CAV is able to inhibit the proliferation of some tumour cell types, such as pancreatic, lung, colon, and leukemic cancers, both *in vitro* and *in vivo* (Green et al., 1980; Swaffar et al., 1994; Thomas et al., 1986). Moreover, L-CAV is cytotoxic for both parental and multidrug-resistant human tumour cells (Worthen et al., 1998). In order to be effective, L-CAV needs to be applied over a long period and at a high dosage, since at low dosages L-CAV may not efficiently compete with L-ARG for incorporation into proteins. Therefore, a clinical potential of L-CAV could best be realized in an adjuvant or drug combination setting.

To date, only one combinatorial study of L-CAV has been carried out. A combination of L-CAV and 5-fluoruracil (5-FU) in human pancreatic carcinoma (MIA PaCa-2) in a molar ratio of 1:1 (L-CAV : 5-FU) in arginine-rich media showed a synergistic effect. An increase of 5-FU in the combination, however, reduced the synergism (Swaffar et al., 1995). A reduction of endogenous L-ARG levels, for example via activation of arginine deiminase (ADI-PEG 20), has been regarded as a novel approach developed to target cancers which are auxotrophic for arginine (Feun et al., 2008). However, arginine deiminase is highly antigenic and has a short half-life, so it needs high dosages to achieve a significant effect. In large amounts, it induces overexpression of argininosuccinate synthase (ASS), a key enzyme in the synthesis of L-ARG in certain melanoma cell lines, leading to drug resistance (Lind, 2004). This study aims to investigate the cytotoxicity of a combination of L-CAV with the chemotherapeutic drugs, doxorubicin (DOX) or cisplatin (CIS), in two types of cancer cell lines, those with ASS overexpression (HeLa and Caco-2 cells) and those with low ASS levels (pancreatic cells MIA PaCa-2 and BxPC-3 cells and hepatocellular carcinoma Hep G2 and SK-HEP-1 cells). Both of the drugs, DOX and CIS are DNA targeting-drugs with different mode of action. DOX has two proposed mechanisms in cancer cells: 1) intercalation into the DNA and disruption of topoisomerase-II mediated DNA repair and 2) production of free radicals, which disturbs cellular membrane, DNA and proteins (Thorn et al., 2011). Unlike DOX, CIS binds to DNA and forms crosslink and DNA adducts. The DNA adduct activates several signalling transduction pathways such as ATR, p53, p73 and MAPK and results in the activation of apoptosis (Florea & Buesselberg, 2011).

One focus of the experiments was to find out whether the combinations of L-CAV with DOX or CIS are additive or synergistic. Our hypothesis is that L-CAV application may result in a synergistic dosage reduction of DOX and CIS in L-ARG deprived cancer cells and enhance their cytotoxicity.

## Methods

### Chemicals

L-Canavanine (purity ≥98%), doxorubicin (≥97%), cisplatin (≥97%), and 3-(4, 5-dimethylthiazol-2-yl)-2,5-diphenyl-tetrazolium bromide (MTT; ≥98%) were purchased from Sigma-Aldrich GmbH, Munich, Germany. DMEM, RPMI 1640, non-essential amino acid (NEAA), sodium pyruvate, penicillin, streptomycin, foetal bovine serum (FBS), trypsin-EDTA, L-glutamine, and dimethylsulfoxide (DMSO) were purchased from Gibco® Invitrogen, Darmstadt, Germany.

### Cell lines and culture condition

We employed the following cancer cell lines in this study: HeLa, Caco-2, MIA PaCa-2, BxPC-3, which were available in our cell culture laboratory (Biology Department, IPMB, Heidelberg), while hepatocellular carcinomas (Hep G2 and SK-HEP-1) were kindly provided by Dr. Kai Breuhahn (Institute of Pathology, Heidelberg). Caco-2 and MIA PaCa-2 cells were maintained in Dulbelcco’s modified Eagle’s medium (DMEM) with Glutamax (Invitrogen/Gibco, Karlsruhe, Germany), supplemented with 10% foetal calf serum (BioChrom KG, Berlin, Germany), 500 U/mL penicillin, 500 μg/mL streptomycin, 1% sodium pyruvate, 1% L-glutamine and 1% NEAA; HeLa and SK-HEP-1 cells were maintained in DMEM media, as mentioned above, without sodium pyruvate and L-glutamine. Hep G2 and BxPC-3 cells were maintained in RPMI 1640 media supplemented with 10% foetal calf serum (FCS), 100 U/mL penicillin, and 100 μg/mL streptomycin. All cells were cultivated at 37 °C, 5% CO_2_, and 95% humidity. For the experiment, 5% dialysed foetal bovine serum (Gibco® Invitrogen, Darmstadt, Germany) were used after exponential growth had been achieved as reported in previous study (Scott et al., 2000).

### Cytotoxicity assay

A dose-dependent cytotoxicity was examined using the MTT assay (Mosmann, 1983). Into each well of 96-well plates, 2 × 10^4^ of cells were seeded, and after 24 h incubation, cells were incubated with test compounds. After 24 h, 0.5 mg/mL of MTT was added to each well of HeLa, Caco-2, MIA PaCa-2, BxPC-3 and SK-HEP-1 cells, while MTT was added to Hep G2 after 48 h incubation with test compounds. The cells were then incubated for 3 h so that the viable cells could produce formazan crystals; they were then dissolved in 100 μL DMSO. After incubation for 10 min in a shaker, the absorption of the formazan was measured at 570 nm using a Tecan Safire II Reader (Tecan Crailsheim, Crailsheim, Germany).

### Cell viability assay

The cell viability of treated cells was examined using the dye exclusion test with trypan blue (Strober, 2001). A cell suspension was mixed with the dye and was visually examined under the microscope within no more than 30 min after treatment. A viable cell showed a translucent cytoplasm compared to a non-viable cell, which showed a blue cytoplasm.

### Combination experiment and analysis of interactions

A combination experiment was conducted using constant and non-constant ratios of cytotoxic agents as developed by Chou (2006). Using a constant ratio, L-CAV was combined with DOX or CIS, based on the IC_50_ value of each drug, IC_70_ of L-CAV and IC_30_ of the chemotherapeutic drug, and one additional combination. These combinations were tested in HeLa, Caco-2, MIA PaCa-2, BxPC-3, Hep G2, and SK-HEP-1 cells. In another set of experiments, a non-constant ratio was used where L-CAV concentrations were maintained at IC_10_ and IC_30_ and then combined with serially diluted DOX or CIS and tested in HeLa, MIA PaCa-2, and SK-HEP-1. The MTT assay was then conducted as outlined above.

Drug interactions were assessed using the combination index method (CI), based on the median-effect principle (Chou, 2006). The median-effect equation correlates the combination of two-drugs and the cytotoxicity effect in the following equation:

\begin{eqnarray*}{{{{({f_a})}_{1,2}}} \over {{{({f_u})}_{1,2}}}} = {{{{({f_a})}_1}} \over {{{({f_u})}_1}}} + {{{{({f_a})}_2}} \over {{{({f_u})}_2}}} - {{{{(D)}_1}} \over {{{({D_m})}_1}}} + {{{{(D)}_2}} \over {{{({D_m})}_2}}}\end{eqnarray*}

When *m* = 1, and

\begin{eqnarray*}{\left[ {{{{{({f_a})}_{1,2}}} \over {{{({f_u})}_{1,2}}}}} \right]^{1/m}} = {\left[ {{{{{({f_a})}_1}} \over {{{({f_u})}_1}}}} \right]^{1/m}} + {\left[ {{{{{({f_a})}_2}} \over {{{({f_u})}_2}}}} \right]^{1/m}} = {{{{(D)}_1}} \over {{{({D_m})}_1}}} + {{{{(D)}_2}} \over {{{({D_m})}_2}}}\end{eqnarray*}

When m ≠ 1

*D* is the dose of the drug, *D_m_* is the median-effect dose signifying the potency, determined from the *x*-intercept of the median-effect plot: *f_a_* is the fraction affected by the dose; *f_u_* is the fraction unaffected (*f_u_* = 1 − *f_a_*); and *m* is an exponent that signifies the sigmoidicity (shape) of the dose-effect curve, which is determined by the slope of the median-effect plot.

The Combination Index (CI) was then calculated using the following equation:

\begin{eqnarray*}{\rm{CI}} = {{{{(D)}_1}} \over {{{({D_x})}_1}}} + {{{{(D)}_2}} \over {{{({D_x})}_2}}}\end{eqnarray*}

The median-effect equation is used to calculate *D_x_*, the dose of drug 1 and 2 alone that inhibits ‘*x*’ percent of cells (IC_50_). (*D*)_1_ and (*D*)_2_ are the concentrations of drug 1 and 2 used in combination to inhibit ‘*x*’ percent of cells (IC_50_).

Type of interaction was analyzed and defined as follow (Table 1) (Chou, 2006):

**Table 1:** Types of interaction of drug combination.

Range of Combination Index	Description	Graded Symbols
<0.1	Very strong synergism	+++++
0.1–0.3	Strong synergism	++++
0.3–0.7	Synergism	+++
0.7–0.85	Moderate synergism	++
0.85–0.90	Slight synergism	+
0.90–1.10	Nearly additive	±
1.10–1.20	Slight antagonism	−
1.20–1.45	Moderate antagonism	−−
1.45–3.3	Antagonism	−−−
3.3–10	Strong antagonism	−−−−
>10	Very strong antagonism	−−−−−

When the drug combination achieved synergy interaction, the dose reduction index (DRI) or cytotoxicity potentiation was calculated to know the fold number or ratio between the concentration of drug alone and the reduced concentration of drug in combination (Chou, 2006) and it is an important issue in clinical level (Eid, El-Readi & Wink, 2012). DRI > 1 indicates synergism. DRI was calculated using the following formula:

\begin{eqnarray*}{\rm{DRI}} = {{{\rm{I}}{{\rm{C}}_{50}}\,{\rm{cytotoxic\; drug\; alone}}} \over {{\rm{I}}{{\rm{C}}_{50}}\,{\rm{cytotoxic\; drug\; in\; combination\; with\; the\; potentiator}}}}\end{eqnarray*}

### Quantitative real-time PCR (qPCR)

qPCR was conducted to examine the ASS expression in 6 different cell lines: HeLa, Caco-2, MIA PaCa-2, BxPC-3, Hep G2, and SK-HEP-1. RNA was extracted from each cell culture; the cultures were grown in 25 cm^2^ culture flasks using RNeasy® Midi Kit (Qiagen, Hilden, Germany) to obtain 500–1000 ng/mL of RNA. A 1.5% gel electrophoresis was used to control the quality of RNA, and a spectrophotometer at OD_260/280_ was used to evaluate the purity and concentration of RNA. cDNA was produced from 1000 ng/mL of RNA using ProtoScript® First Strand cDNA Synthesis Kit (New England BioLabs, Inc, Ipswich, MA, USA). Amplification of 1/10 of these cDNA by qPCR was performed using the following gene-specific primers: ASS sense (5′- CAG ACG CTA TGT CCA GCA AA-3′) and ASS antisense (5′- TGC TTT GCG TAC TCC ATC AG-3′). Glyceraldehyde-3-phosphatase dehydrogenase (G-3-PDH) was used as a reference gene and was assessed using the following gene-specific G-3-PDH primers: G-3-PDH sense (5′- GAA CAT CAT CCC TGC CTC TAC TG–3′) and G-3-PDH antisense (5′-GTT GCT GTA GCC AAA TTC GTT G–3′). PCR amplifications were carried out using qTOWER Real-Time PCR Thermal Cycler (Analytik Jena AG, Jena, Germany), with the following temperature cycling parameters 94 °C/45 s; 65 °C/2 min; 72 °C/2 min for 45 cycles; and a final extension at 72 °C/10 min. The analysis of gene expression data from qPCR experiment and ASS expression was performed as a function of 2^-ΔΔCt, in which the Ct value from each cell is compared to the Ct value from the cell with the highest Ct value (lowest ASS expression) and GAPDH expressions are not significantly changed.

### Statistical analysis

All tests were performed in triplicate and repeated at least three times. All data are expressed as a mean ± standard deviation. All *p* values were calculated using Student’s t-test. The difference was considered to be statistically significant at the level of *p* < 0.05. The IC_50_ values were calculated from the dose-response curves using a four-parameter logistic fitting curve (SigmaPlot® 11.0). GraphPad Prism® software (Graphpad Prism® 5.01, GraphPad Software, Inc, CA, USA) was used to draw the graphs.

## Results

### Expression levels of ASS

**Figure 1:**
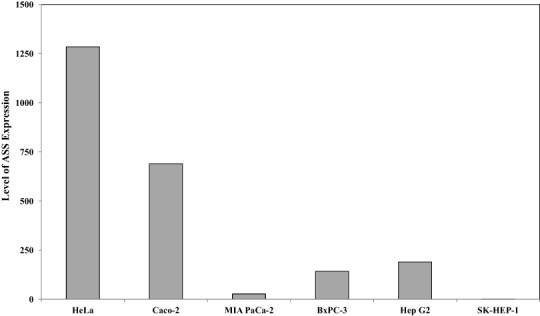
Relative argininosuccinate synthase (ASS) expression in HeLa, Caco-2, MIA PaCa-2, BxPC-3, Hep G2, and SK-HEP-1 in which GAPDH expressions are not significantly changed. Data from three independent experiments are represented as mean ± SD.

Quantitative real time PCR was performed to evaluate the expression of argininosuccinate synthase (ASS) at the mRNA level in the 6 cancer cell lines used in this study. Figure 1 illustrates that HeLa and Caco-2 expressed higher ASS levels as compared with pancreatic cells (MIA PaCa-2 and BxPC-3) and hepatocellular carcinoma (Hep G2 and SK-HEP-1). SK-HEP-1 expressed the lowest level of ASS among 5 other cell lines. This finding is in agreement with other studies using RT-PCR and western blot analysis showing that pancreatic and hepatocellular cancer cells express low levels of ASS (Bowles et al., 2008; Feun et al., 2008; Liu et al., 2014).

### The effect of L-ARG deprivation on the cytotoxicity of DOX or CIS

In order to find out whether L-ARG deprivation in the cell culture medium significantly affects the activity of DOX and CIS, the cytotoxicity of DOX and CIS as single substances was determined in L-ARG-free media (AFM) and L-ARG-rich media (ARM) in 6 cancer cell lines. Table 2 shows that L-ARG deprivation caused significant differences in the IC_50_ of DOX and CIS in HeLa, Bx-PC3, Hep G2 and SK-HEP-1 cells as compared to those in normal media. However, no significant difference was observed in Caco-2 and MIA PaCa-2 (t-test analysis with *p* <0.05 indicating significant differences). L-CAV itself is only slightly cytotoxic for the 6 cancer cell lines grown in L-ARG-free media with IC_50_ in the range of 0.2–1.2 mM (Tables 3 and 4) as has been shown in other studies (Jang et al., 2002; Swaffar et al., 1995).

**Table 2:** IC_50_ values (μM) of doxorubicin (DOX) and cisplatin (CIS) in arginine-rich media (ARM) and arginine-free media (AFM).

N°	Cells	DOX (IC_50_ ± SD)		CIS (IC_50_ ± SD)	
		ARM	AFM	ARM	AFM
1.	HeLa	1.66 ± 0.30^*^	4.07 ± 0.26^*^	77.36 ± 9.11^*^	54.07 ± 12.25^*^
2.	Caco-2	33.55 ± 4.93	34.91 ± 5.70	105.88 ± 14.66	96.38 ± 32.03
3.	MIA PaCa-2	14.60 ± 1.21	22.25 ± 3.35	72.01 ± 3.50	97.20 ± 20.34
4.	BxPC-3	5.00 ± 0.74^*^	6.90 ± 0.53^*^	126.42 ± 34.07^*^	85.66 ± 3.81^*^
5.	Hep G2	1.86 ± 0.38^*^	4.68 ± 1.08^*^	23.99 ± 2.75^*^	14.87 ± 1.22^*^
6.	SK-HEP-1	7.92 ± 2.43^*^	11.09 ± 0.82^*^	117.40 ± 15.72	77.89 ± 17.77

**Note:**

Data from three independent experiments (n = 3) are represented as mean ± SD. The asterisk indicates a significant difference (at *P* < 0.05 analyzed using t-Test) in the cytotoxicity of the chemotherapeutic drug in cancer cells treated with arginine rich media (ARM) compared with arginin free media (AFM).

**Table 3:** IC_50_ values (μM) of doxorubicin (DOX) alone and in combination with L-canavanine (L-CAV) (constant ratio).

N°	Cells	Drug alone				Combination of L-CAV and DOX				
		L-CAV		DOX		Combination (L-CAV : DOX)	Molar ratio (L-CAV : DOX)	DOX (IC_50_ ± SD)	CI Value	Interaction
		(IC_50_ ± SD)	(IC_70_ ± SD)	(IC_50_ ± SD)	(IC_30_ ± SD)					
						10 : 1	10 : 1	0.53 ± 0.03	0.1547	++++
1.	HeLa	216.9 ± 47.72	581.41 ± 106.99	4.07 ± 0.26	2.92 ± 0.65	IC_50_ : IC_50_	53.29 : 1	0.13 ± 0.02	0.0644	+++++
						IC_70_ : IC_30_	199.11 : 1	0.19 ± 0.07	0.2220	++++
						5 : 1	5 : 1	11.16 ± 2.79	0.4215	+++
2.	Caco-2	589.60 ± 138.92	1867.27 ± 623.99	34.91 ± 5.70	23.13 ± 2.46	IC_50_ : IC_50_	17.25 : 1	1.32 ± 0.32	0.0790	+++++
						IC_70_ : IC_30_	80.73 : 1	1.12 ± 0.05	0.1727	++++
						5 : 1	5 : 1	11.09 ± 2.41	0.5605	+++
3.	MIA PaCa-2	865.50 ± 315.30	3515.56 ± 978.67	22.25 ± 3.35	17.27 ± 3.04	IC_50_ : IC_50_	38.89 : 1	3.65 ± 0.61	0.3434	+++
						IC_70_ : IC_30_	203.56 : 1	2.34 ± 0.15	0.7562	++
						100 : 1	100 : 1	2.44 ± 0.29	0.4656	+++
4.	Bx-PC 3	2167 ± 282.84	3563.97 ± 345.07	6.90 ± 0.53	3.25 ± 0.36	IC_50_ : IC_50_	328.33 : 1	2.06 ± 0.08	0.6130	+++
						IC_70_ : IC_30_	1096.60 : 1	0.81 ± 0.18	0.6573	+++
						50 : 1	50 : 1	1.67 ± 0.28	0.4328	+++
5.	Hep G2	640.90 ± 164.68	1202.75 ± 397.31	4.68 ± 1.08	1.19 ± 0.39	IC_50_ : IC_50_	149.74 : 1	1.27 ± 0.30	0.4602	+++
						IC_70_ : IC_30_	1010.71 : 1	0.55 ± 0.05	0.9874	±
						10 : 1	10 : 1	3.03 ± 0.25	0.2989	++++
6.	SK-HEP-1	1179 ± 149.08	2018.37 ± 678.23	11.09 ± 0.82	3.75 ± 0.73	IC_50_ : IC_50_	105.36 : 1	2.25 ± 0.02	0.3985	+++
						IC_70_ : IC_30_	538.23 : 1	0.42 ± 0.06	0.2296	++++

**Note:**

Data from three independent experiments (n = 3) are represented as mean ± SD.

**Table 4:** IC_50_ values (μM) of cisplatin (CIS) alone and in combination with L-canavanine (L-CAV) (constant ratio).

N°	Cells	Drug alone				Combination of L-CAV and DOX				
		L-CAV		CIS		Combination (L-CAV : CIS)	Molar ratio (L-CAV : CIS)	CIS (IC_50_ ± SD)	CI Value	Interaction
		(IC_50_ ± SD)	(IC_70_ ± SD)	(IC_50_ ± SD)	(IC_30_ ± SD)					
						50 : 1	50 : 1	0.60 ± 0.13	0.1875	++++
1.	HeLa	216.9 ± 47.72	581.41 ± 106.99	54.07 ± 12.25	34.24 ± 8.82	IC_50_ : IC_50_	4.01 : 1	3.58 ± 0.39	0.1359	++++
						IC_70_ : IC_30_	16.98 : 1	1.29 ± 0.24	0.1275	++++
						100 : 1	100 : 1	1.89 ± 0.65	0.2200	++++
2.	Caco-2	589.60 ± 138.92	1867.27 ± 623.99	96.38 ± 32.03	46.18 ± 13.40	IC_50_ : IC_50_	6.11 : 1	4.79 ± 0.06	0.1195	++++
						IC_70_ : IC_30_	40.44 : 1	3.00 ± 0.83	0.2606	++++
						5 : 1	5 : 1	55.34 ± 2.73	0.8887	++
3.	MIA PaCa-2	865.50 ± 315.30	3515.56 ± 978.67	97.20 ± 20.34	41.70 ± 10.02	IC_50_ : IC_50_	9.40 : 1	56.36 ± 1.34	1.1970	−
						IC_70_ : IC_30_	84.31 : 1	3.87 ± 0.17	0.5018	+++
						10 : 1	10 : 1	57.68 ± 5.04	0.9041	+
4.	BxPC-3	2167 ± 282.84	3563.97 ± 345.07	85.66 ± 3.81	48.34 ± 5.44	IC_50_ : IC_50_	24.59 : 1	18.06 ± 2.09	0.4032	+++
						IC_70_ : IC_30_	73.72 : 1	6.79 ± 0.48	0.2394	++++
						10 : 1	10 : 1	11.16 ± 3.14	0.6425	+++
5.	Hep G2	640.90 ± 164.68	1202.75 ± 397.31	14.87 ± 1.22	7.42 ± 0.24	IC_50_ : IC_50_	43.10 : 1	8.01 ± 0.53	0.8749	+
						IC_70_ : IC_30_	162.09 : 1	2.02 ± 0.03	0.5949	+++
						100 : 1	100 : 1	4.28 ± 0.46	0.4181	+++
6.	SK-HEP-1	1179 ± 149.08	2018.37 ± 678.23	77.89 ± 17.77	51.38 ± 16.26	IC_50_ : IC_50_	15.14 : 1	13.72 ± 2.87	0.3070	+++
						IC_70_ : IC_30_	39.28 : 1	7.34 ± 0.27	0.2806	++++

**Note:**

Data from three independent experiments (n = 3) are represented as mean ± SD.

### Combinations of L-CAV with DOX or CIS

L-CAV was combined with DOX or CIS using two different approaches, i.e., in constant or non-constant ratios, as developed by Chou (2006). Constant ratio combinations were carried out employing three different molar ratios of L-CAV and DOX or CIS. The first molar ratio of combination was based on the IC_50_ value of each drug; the second was chosen by increasing the toxicity of L-CAV to the value of IC_70_ and decreasing the toxicity of DOX or CIS to the value of IC_30_; and the third one was an additional combination designed to observe the interaction pattern of a combination according to the presence of L-CAV. For non-constant ratios a constant non-toxic value of IC_10_ or IC_30_ of L-CAV was employed, while the concentration DOX or CIS covered the whole concentration range.

**Figure 2:**
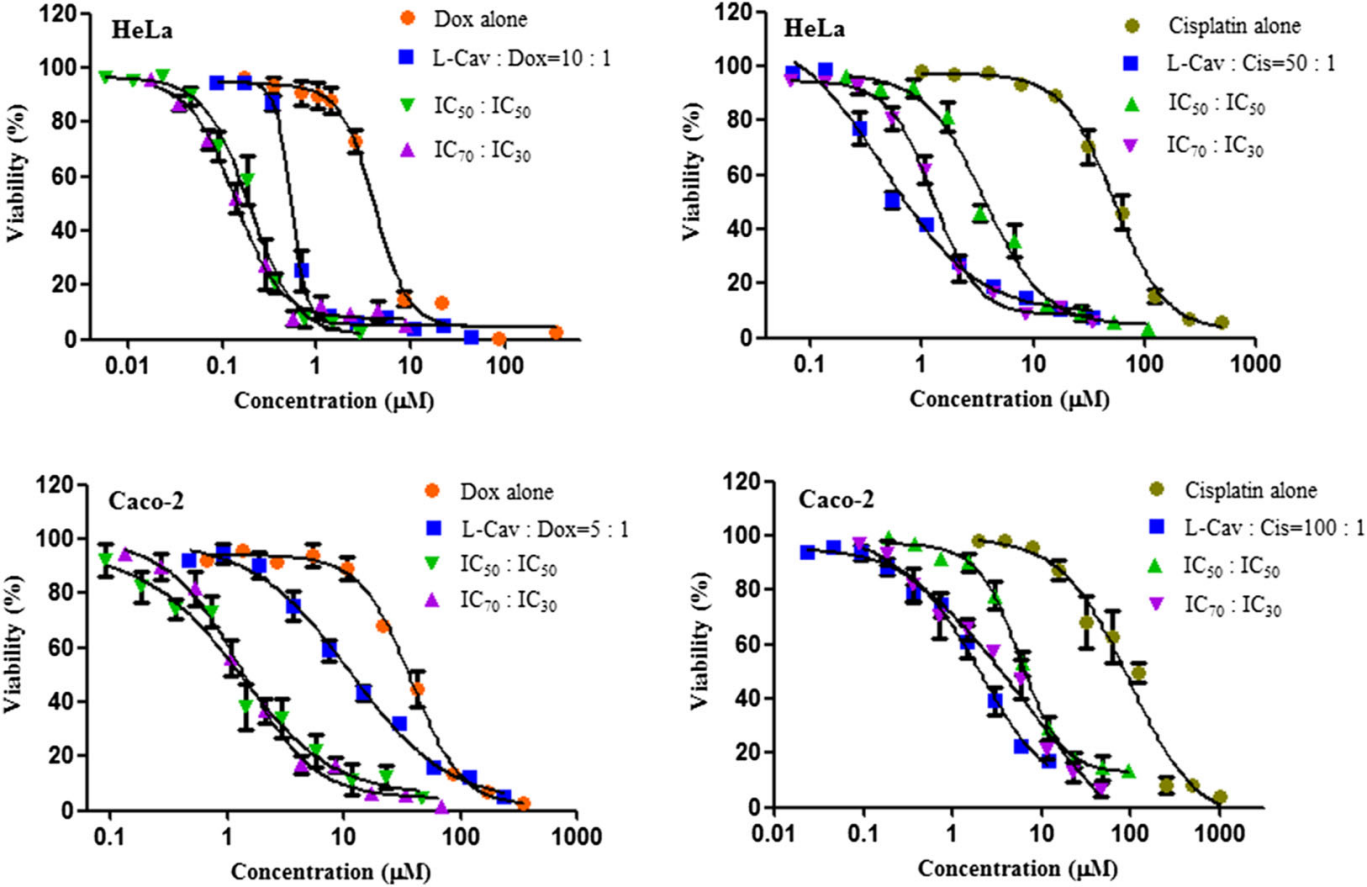
Dose response curves of the combination of L-canavanine (L-CAV) and doxorubicin (DOX), and L-canavanine (L-CAV) and cisplatin (CIS) for the growth inhibition of HeLa and Caco-2, using a constant ratio method in arginine-free media (AFM). Data from three independent experiments are represented as mean ± SD.

**Figure 3:**
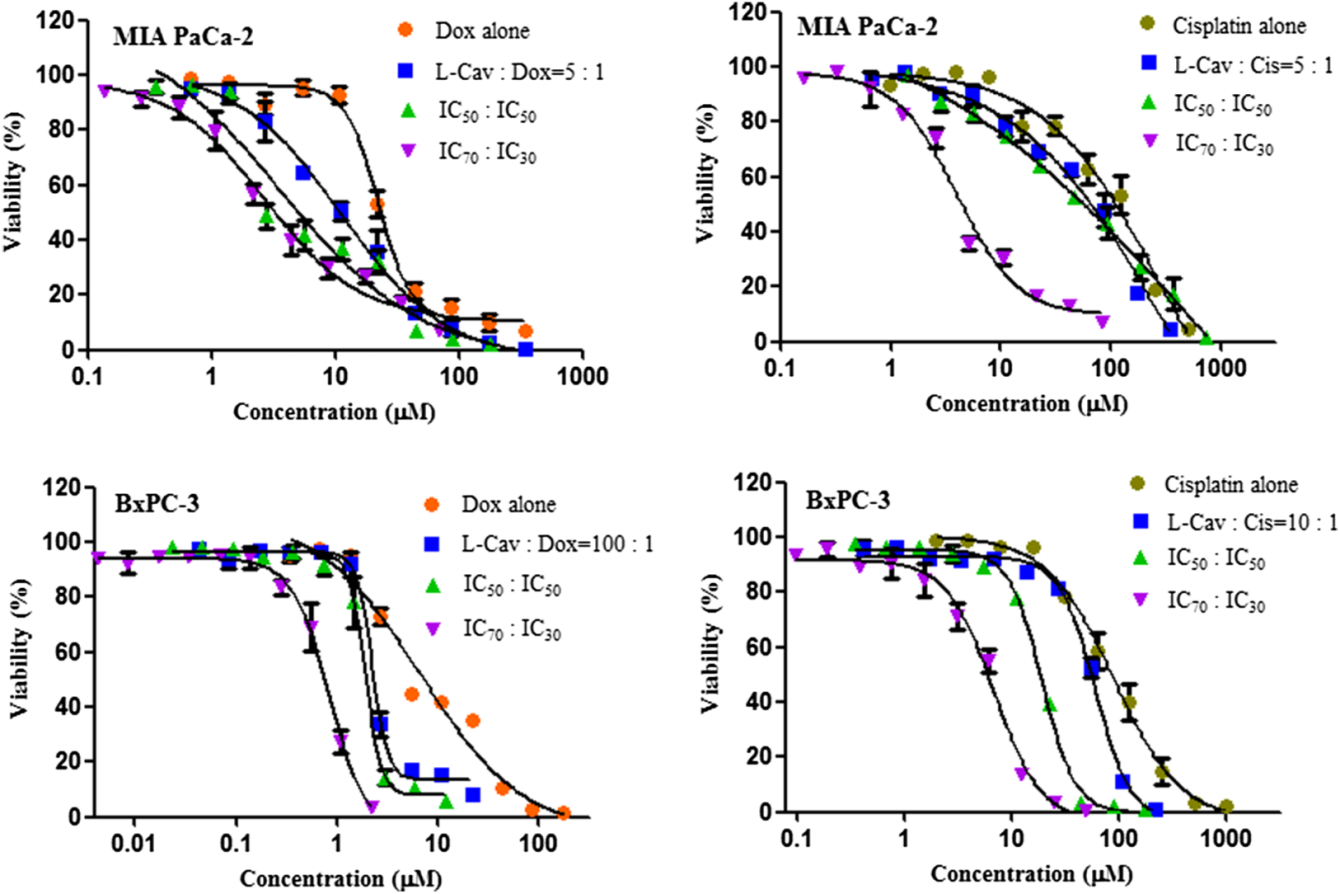
Dose response curves of the combination of L-canavanine (L-CAV) and doxorubicin (DOX), and L-canavanine (L-CAV) and cisplatin (CIS) for the growth inhibition of MIA PaCa-2 and BxPC-3, using a constant ratio method in arginine-free media (AFM). Data from three independent experiments are represented as mean ± SD.

**Figure 4:**
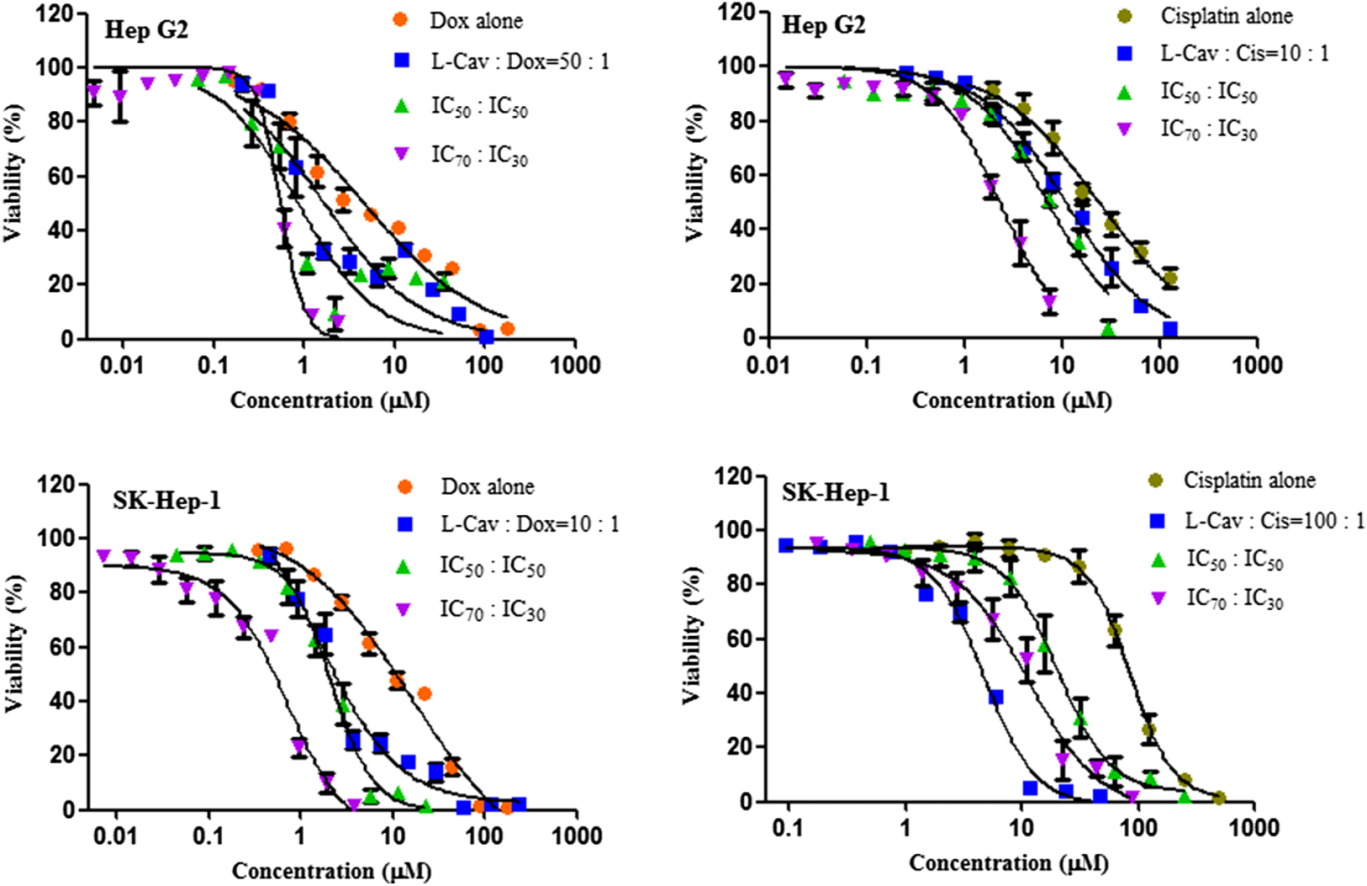
Dose response curves of the combination of L-canavanine (L-CAV) and doxorubicin (DOX), and L-canavanine (L-CAV) and cisplatin (CIS) for the growth inhibition of Hep G2 and SK-Hep-1, using a constant ratio method in arginine-free media (AFM). Data from three independent experiments are represented as mean ± SD.

Figures 2, 3, and 4 illustrates the results of the combination of L-CAV with DOX and CIS, whose cytotoxicity could be substantially enhanced by all combination treatments. Detailed results of these combinations are documented in Table 3 (DOX) and Table 4 (CIS). The combination of L-CAV and DOX (both IC_50_ concentration) is not significantly different to the combination in which the IC_70_ dosage of L-CAV and IC_30_ dosage of DOX were employed in HeLa, Caco-2, MIA PaCa-2, and BxPC-3 cells. However, a significant difference was observed in both hepatocellular carcinomas, Hep G2 and SK-HEP-1 cells (Table 3). When L-CAV was combined with CIS, IC_50_ and IC_70_ concentrations of L-CAV were significantly different in the cell lines (except SK-HEP-1), as summarized in Table 4.

In order to interpret the nature of combinations, the corresponding CI values were calculated (Tables 3 and 4). The combinations of L-CAV with DOX were synergistic in the six cell lines; only the IC_70_:IC_30_ combination of L-CAV with DOX in Hep G2 cells showed a nearly additive effect (Table 3). The combinations of L-CAV with CIS are illustrated in Table 4. Most of the combinations are synergistic, with the exception of the IC_50_:IC_50_ combination in MIA PaCa-2 cells, which suggested a slight antagonism.

In a second set of experiments with HeLa (overexpressing ASS) and MIA PaCa-2 and SK-HEP-1 cells (low ASS expression), a non-constant ratio was used in which L-CAV was applied at a constant IC_10_ or IC_30_ value, whereas DOX and CIS were serially diluted. Table 5 is a summary of these combinations, clearly showing that all combinations were synergistic in nature.

**Table 5:** IC_50_ values (µM) of doxorubicin (DOX) and cisplatin (CIS) alone and in combination with L-canavanine (L-CAV), using non-constant ratio combinations.

N°	Cell	IC_50_ value of DOX or CIS Alone (IC_50_ ± SD)	Combination	IC_50_ value of DOX or CIS in Combination (IC_50_ ± SD)	CI	Interpretation
1	HeLa	4.07 ± 0.26	IC_10_ of L-CAV and DOX	0.11 ± 0.03	0.11	Strong synergism
2			IC_30_ of L-CAV and DOX	0.0043 ± 0.0015	0.38	Synergism
3		54.07 ± 12.25	IC_10_ of L-CAV and CIS	0.30 ± 0.09	0.09	Very strong synergism
4			IC_30_ of L-CAV and CIS	0.066 ± 0.02	0.38	Synergism
5	MIA PaCa-2	22.25 ± 3.35	L-CAV IC_10_ and DOX	2.44 ± 0.28	0.14	Strong synergism
6			L-CAV IC_30_ and DOX	0.62 ± 0.06	0.27	Strong synergism
7		97.20 ± 20.34	L-CAV IC_10_ and CIS	88.80 ± 5.44	0.81	Moderate synergism
8			L-CAV IC_30_ and CIS	14.57 ± 2.85	0.37	Synergism
9	SK-HEP-1	11.09 ± 0.82	L-CAV IC_10_ and DOX	0.32 ± 0.09	0.28	Strong synergism
10			L-CAV IC_30_ and DOX	0.31 ± 0.08	0.61	Synergism
11		77.89 ± 17.77	L-CAV IC_10_ and CIS	20.72 ± 2.16	0.44	Synergism
12			L-CAV IC_30_ and CIS	4.56 ± 1.31	0.63	Synergism

**Note:**

Data from three independent experiments (n = 3) are represented as mean ± SD.

**Figure 5:**
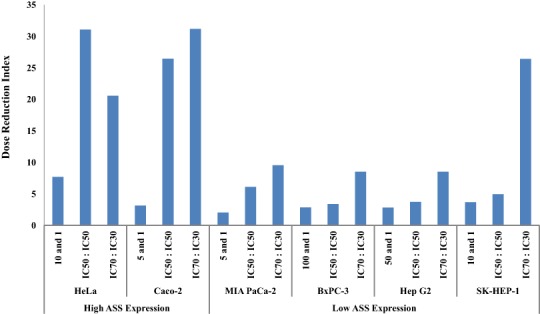
Dose Reduction Index (DRI) of doxorubicin (DOX). L-Canavanine (L-CAV) was combined with DOX using a constant ratio in two types of cells, cells with high ASS expression (HeLa and Caco-2) and cells with low ASS expression (MIA PaCa-2, BxPC-3, Hep G2, and SK-HEP-1).

**Figure 6:**
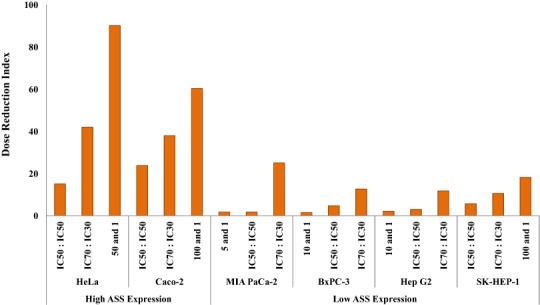
Dose Reduction Index (DRI) of cisplatin (CIS). L-Canavanine (L-CAV) was combined with CIS using a constant ratio in two types of cells, cells with high ASS expression (HeLa and Caco-2) and cells with low ASS expression (MIA PaCa-2, BxPC-3, Hep G2, and SK-HEP-1).

The dosage reduction index (DRI) of DOX and CIS, calculated from 6 different cell lines, is illustrated in Figs. 5 and 6. The higher DRI value for HeLa and Caco-2 cells indicates that both cell lines are more sensitive to the combination treatment as compared with pancreatic cells (MIA PaCa-2 and BxPC-3) and hepatocellular carcinoma cells (Hep G2 and SK-HEP-1). A dosage reduction of CIS was observed to be superior as compared with DOX in some cancer cell lines.

## Discussion

Based on the mode of action, L-CAV is categorized as an antimetabolite, as it inhibits the metabolism of arginine (L-ARG), to which it is structurally related. The only difference between these two structures is the replacement of the methylene bridge (-CH2- unit) in L-ARG with an oxygen in L-CAV, making it less basic. L-CAV can be incorporated into the protein because arginyl-tRNA synthetase is unable to distinguish between L-ARG and L-CAV. Some studies have reported that L-CAV replaces L-ARG in the protein in Walker carcinoma 256 cells (Kruse et al., 1959), Chinese hamster ovary cells (Laszlo & Li, 1993), HeLa S-3 cells (Wheatley & Robertson, 1981), and Hep G2 cells (Redman, Avellino & Yu, 1983), and that an incorporation leads to the alteration of protein conformation (Rosenthal, Reichhart & Hoffmann, 1989) in murine leukemia virus, moloney murine sarcoma virus (Murphy & Arlinghaus, 1980), and Hep G2 cells (Redman, Avellino & Yu, 1983). However, because L-CAV is structurally related to L-ARG, a high concentration of L-CAV is required to outcompete L-ARG, which is normally present as an essential amino acid in good quantities.

In this study, to enhance the efficacy of L-CAV, the concentration of L-ARG was reduced in the cell culture media. In clinical application, deprivation of L-ARG can be achieved via arginine deiminase (ADI) which has high affinity to arginine and catalyzes arginine to citrulline and ammonia. The application of L-ARG deprivation has been optimized using polyethylene glycol (PEG) technology to diminish immunogenicity, increase bioavailability, and selectively degrade arginine, therefore resulting in tumour cell death (Feun et al., 2008). In phase I/II clinical trials, a weekly schedule of intramuscular application of ADI-PEG 20 resulted in 25% and 47% (complete and partial) response in patients with advanced melanoma and hepatocellular carcinoma (Izzo et al., 2004; Ascierto et al., 2005). Clinical trial with several hundred patients with cancer has revealed that ADI-PEG 20 is feasible and safe. However, a concern shall be taken in several patients in whom arginine deprivation can cause contraindication related to T cell immunity and cardiac function, such as in patients with an urea-cycle disorders, active or treated infections (TB and HIV), immunodeficiency and cardiovascular disease (Delage et al., 2010). In addition, arginine deprivation can also induce ASS expression in certain melanoma cell lines which possibly lead to drug resistance (Feun et al., 2008; Savaraj et al., 2007). Combination of L-ARG deprivation (i.e. ADI) with other cytotoxic agent or targeted agent can reduce the side effect, for instances hyperuricaemia in the case of hepatocelular carcinoma, other side effects such as pain in the injection side and also the possibility of drug resistance (Delage et al., 2010). Combination of L-ARG deprivation with L-ARG analogue, L-CAV is a rational approach. In addition the combination with chemotherapeutic drugs, such as DOX and CIS will enhance the anticancer activity while reducing the toxicity and side effects.

Application of L-CAV may compete with metabolic reactions in which arginine is the substrate of reaction, such as polyamine synthesis. A study using rat bearing colon tumour treated with L-CAV alone showed a body weight loss after certain dose and schedule of administration (Thomas et al., 1986). Combination of L-CAV with other agents could reduce the L-CAV dose, diminish the body weight loss (Thomas and Rosenthal, 1987) and reduce the possible immunotoxic effect, as seen in peripheral blood mononuclear cells (Bence et al., 2002). The potency of L-CAV in drug combination, for instance with other chemotherapeutic drugs is still little studied. Only a study conducted by Swaffar and co-workers has reported a potential combination of L-CAV and classical antimetabolite, 5-fluoruracil in human pancreatic cancer cells (Swaffar et al., 1995).

In this study, drug combinations were evaluated in 6 different cancer cell lines, which differed in the expression of argininosuccinate synthase (ASS). Some studies have investigated the importance of ASS deficiency and the effect of L-ARG deprivation in human cancers (Bowles et al., 2008; Dillon et al., 2004; Scott et al., 2000; Sugimura et al., 1992). As confirmed in this study, HeLa and Caco-2 cells exhibit a higher ASS expression as compared to both pancreatic cancer cells (MIA PaCa-2 and BxPC-3) and hepatocellular carcinoma cells (Hep G2 and SK-HEP-1) (Fig. 1). The cytotoxicity of DOX was found to be lower in HeLa, Bx-PC3, Hep G2 and SK-HEP-1 treated in AFM as compared to ARM. Deprivation of ARG concentration in the culture media of HeLa, Bx-PC3, Hep G2 and SK-HEP-1 disturbed cell metabolism and may affect the active transport protein, which related to the reduction of uptake kinetic to DOX, known as a drug substrate, belonging to the adenosine triphosphate (ATP) binding cassette (ABC) transporters (Katayama, Noguchi & Sugimoto, 2014). Thus, a reduction of DOX cytotoxicity was observed in those cells with ARG deprivation. On the other hand, an increased cytotoxity of CIS was noted in HeLa, Bx-PC3, Hep G2 and SK-HEP-1 when the cells were treated in AFM as compared to ARM. Some authors have reported that cisplatin resistance in some cancer cells is due to the rapid efflux of CIS (Wang & Lippard, 2005). This result suggests that L-ARG deprivation alone is not sufficient to enhance the cytotoxicity of DOX and CIS. But L-CAV could potentiate the cytotoxicity of DOX and CIS, especially in cells expressing high levels of ASS as compared to the cells with reduced ASS levels (Tables 3 and 4).

How can this difference be explained? Cells expressing ASS also show high levels of arginyl-tRNA synthetase (Kim, You & Hwang, 2011). Arginyl-tRNA synthetase is an enzyme which uses L-ARG as a substrate to form the complex L-arginyl-tRNA(Arg), necessary in ribosomal protein synthesis. L-CAV acts as an alternative substrate for arginyl-tRNA synthetase; thus, in cells expressing high ASS, higher levels of arginyl-tRNA synthetase may increase the chance of L-CAV incorporation into a protein in place of L-ARG. Enzymes which become functionless may include the battery of DNA repair enzymes or proteins involved in drug metabolism (including ABC transporters), which often reduce the toxicity of chemotherapeutic drugs. As a consequence, such tumour cells will become more sensitive to DOX and CIS when treated with L-CAV in combination.

In Caco-2 and MIA PaCa-2, the cytotoxicity of DOX and CIS in IC_50_ values did not show a significant difference in ARM and AFM. When a combination of L-CAV and DOX or CIS is performed in ARM, we assume that L-CAV can potentiate the cytotoxicity of drugs in both cell lines, optimally using sequence–dependent administration (L-CAV following the chemotherapeutic drugs) instead of simultaneous-dependent administration as used in this study. Administration of L-CAV at the first order for a certain period may efficiently compete with the presence of L-ARG and resulting the sensitivity of cancer cells to the chemotherapeutic drugs.

Scientific reason why different tumor cell lines possess different levels of ASS expression remains unclear. Previous studies indicate that ASS regulation occurs at pre-translational levels (Husson et al., 2003) and it can be influenced by multiple factors and may be tissue specific. Further investigation is necessary to identify the dose reduction index in the cells where ASS activity is decreased. For instance, using pharmacological inhibition of ASS such as fatty acids; insulin and growth hormone in liver tissue (Feun et al., 2008) or by silencing of ASS expression to understand in detail the role of ASS protein and related signalling pathway that can be influenced due to the application of L-CAV.

## Conclusions

In conclusion, the mechanism underlying the synergistic interaction between L-CAV and two DNA interacting drugs could be explained by L-ARG deprivation, which facilitates L-CAV incorporation into proteins in place of L-ARG. Incorporation of L-CAV disturbs the correct 3D-structure of target proteins and thus leads to protein dysfunction or prevention of repair of DNA damage. This condition may sensitize cancer cells for DOX and CIS. This study has revealed an interesting potentiation of L-CAV in combination with chemotherapeutic drugs, specific DNA-targeting drugs in L-ARG auxotrophic and non-auxotrophic cancer cell lines.

*In vivo* study employing an exact dose level and schedule of administration in combination with the chemotherapeutic drugs is warranted to understand whether drug combination works *in vivo*. The mechanism of combination, complex protein signaling pathways and virtually metabolic reactions associated with L-ARG utilizing pathway requires a further investigation.

## Supplemental Information

10.7717/peerj.1542/supp-1 Supplemental Information 1 L-Canavanine potentiates the cytotoxicity of doxorubicin and cisplatin in arginine deprived human cancer cells (Raw data). L-Canavanine (L-CAV) was combined with doxorubicine (DOX) or cisplatin (CIS) and then tested against several human cancer cells: human cervical cancer (HeLa), colon cancer (Caco-2), pancreatic cancer cell (MIA PaCa-2 and Bx-PC3), and hepatocellular carcinoma (Hep G2 and SK-Hep-1) in arginine free media (AFM). Cytotoxicity of a single drug was also examined in arginine rich media (ARM). Three types of constant ratio combinations were employed, those are combination based on IC50 of each drug (IC50 : IC50), IC70 of L-CAV and IC30 of drug (IC70 : IC30) and an extra combination to understand whether the combination works in L-CAV dose-dependent manner. Analysis of combination interaction was determined based on combination index (CI) method. Dose reduction index (DRI) was determined to identify the fold number or ratio between the concentration of drug alone and the reduced concentration of the drug in combination in which it is an important issue in clinical level.

## Additional Information and Declarations

### Data Deposition

The following information was supplied regarding data availability: The raw data is supplied as a Supplemental Dataset file.
